# A squash and a squeeze

**DOI:** 10.7554/eLife.80416

**Published:** 2022-06-30

**Authors:** Danelle Devenport

**Affiliations:** 1 Department of Molecular Biology, Princeton University Princeton United States

**Keywords:** morphogenesis, visceral organ, light sheet microscopy, mesoderm, calcium signaling, hox genes, *D. melanogaster*

## Abstract

Advanced imaging techniques reveal details of the interactions between the two layers of the embryonic midgut that influence its ultimate shape.

**Related research article** Mitchell NP, Cislo DJ, Shankar S, Lin Y, Shraiman BI, Streichan SJ. 2022. Visceral organ morphogenesis via calcium-patterned muscle constrictions. *eLife* **11**:e77355. doi: 10.7554/eLife.77355.

The gastrointestinal tract of most animal species is far longer than the body in which it is housed. The human gut, for example, is approximately 20 feet long and must fold, loop and twist to fit inside the body (Helander and Fändriks, 2014). Remarkably, contortions of the gut tube are highly stereotyped and species-specific, indicating that the formation of the folds is genetically controlled (Savin et al., 2011). However, it remains unclear how the instructions encoded within the genome lead to such precise and reproducible changes of shape.

To get to the bottom of this, developmental biologists seek to describe the movements of individual cells and connect these to a change in the shape of the whole organ. This remains a challenge, especially for internal organs, which develop deep within embryos and whose shape is determined by the interactions between multiple layers of tissue. Recent advances in live imaging using 3D light-sheet microscopy have allowed biologists to visualize morphological change on the surface of whole embryos, but internal organs like the gut have remained largely out of reach (Wan et al., 2019).

Now, in eLife, Sebastian Streichan of the University of California Santa Barbara and colleagues – including Noah Mitchell as first author – report a new method that combines deep-tissue light-sheet microscopy with a framework to analyze shape changes between tissue layers in the gut of fruit flies (Mitchell et al., 2022).

The midgut of fruit flies begins as a simple tube consisting of an inner epithelial layer ensheathed by smooth muscle. The gut tube then constricts at three precise positions, which subdivides the tube into four chambers as it changes shape and gets longer (Figure 1). To visualize this folding and elongation, Mitchell et al. expressed fluorescent markers selectively in cells of the midgut and used genetically modified, transparent embryos to reduce light scatter. Using confocal multiview light-sheet microscopy, the researchers generated time-lapse movies of full, 3D volumes of the developing midgut (de Medeiros et al., 2015). By measuring the geometry of whole organs, they found that the length of the gut tube triples during folding, while maintaining a near constant volume. This occurs in the absence of cell divisions, suggesting that changes in the shape of cells may be responsible for the elongation.

**Figure 1.**
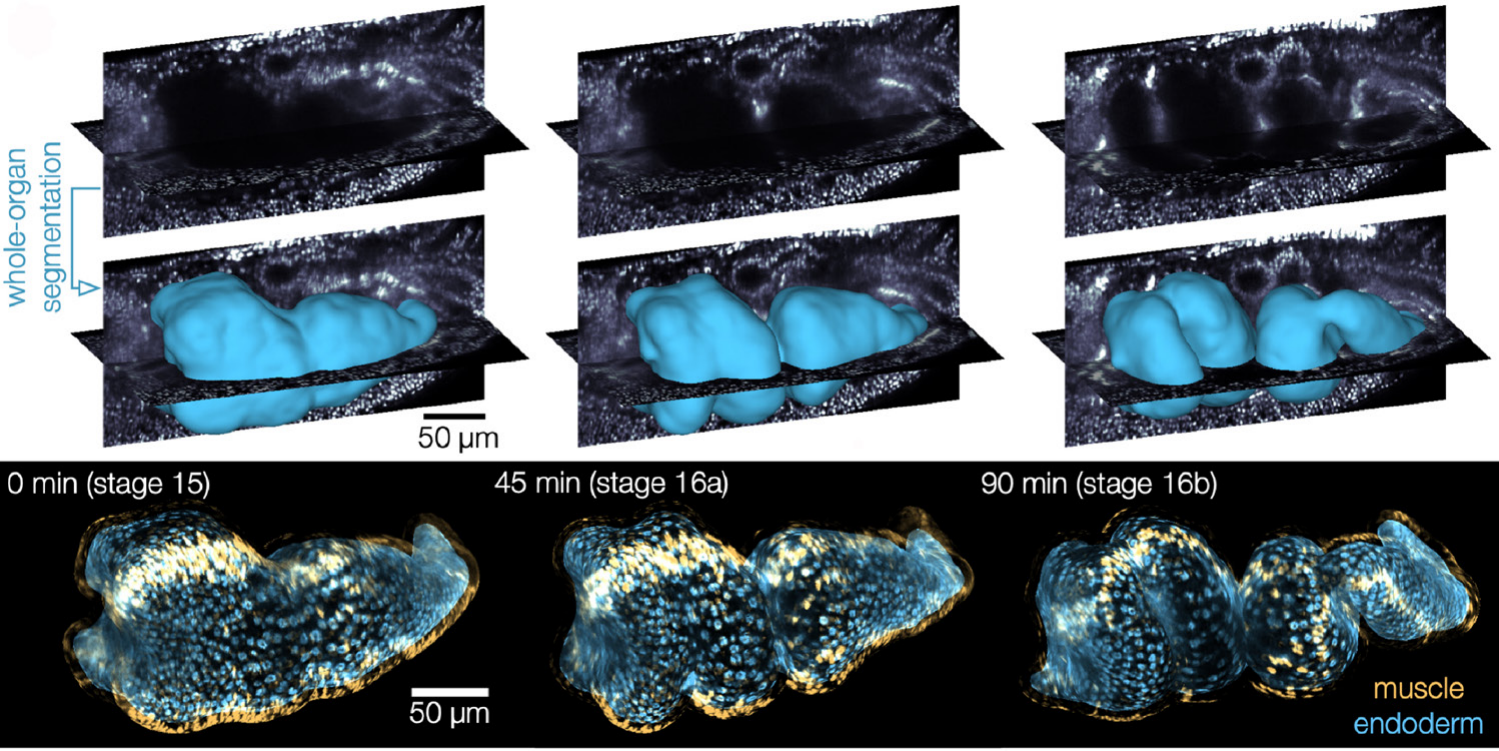
Shaping of the developing midgut of fruit flies. Top: Automatic segmentation tools enable layer-specific imaging of the muscle (yellow ) and endoderm (blue) to generate a 3D shape. Bottom: The midgut initially consists of muscle cells (yellow) and a layer of endodermal cells (blue), which interact to mold the gut into shape. The gut tube constricts at three precise positions, which subdivide it into four chambers before it starts to coil.

To find out how the behavior of individual cells drives the constriction and elongation of the gut, Mitchell et al. developed an image analysis package aptly named TubULAR. This programme combines machine learning and computer vision techniques that link cell movements to changes in the shape of the whole organ. In many epithelia, cell intercalations – a process during which neighboring cells switch places – drive tissue convergence and extension (Paré and Zallen, 2020; Sutherland et al., 2020). In the gut tube, however, constriction and elongation correlated with patterned changes in the epithelial cell shape (Figure 2A and B).

**Figure 2.**
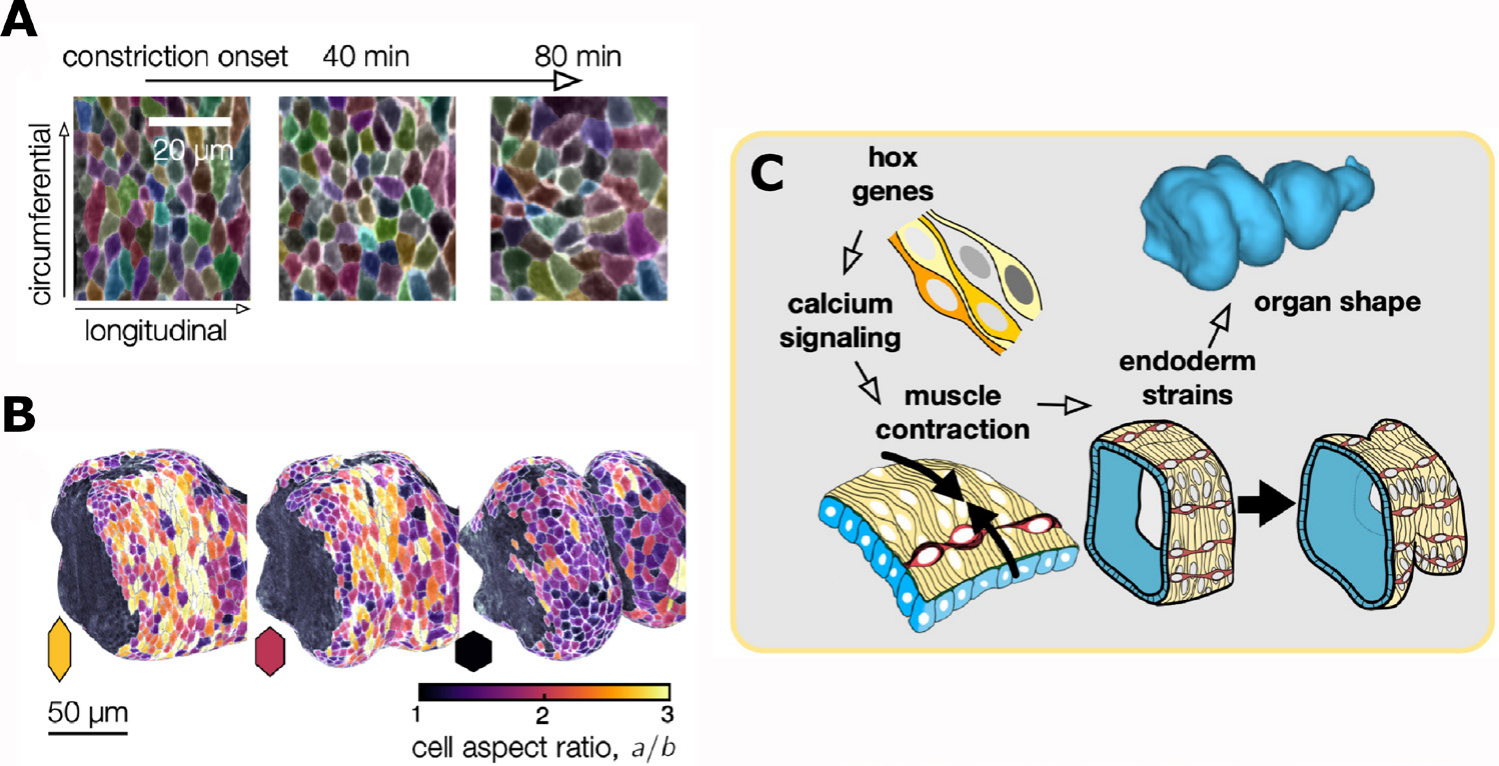
Changes in the shape of endodermal cells are linked to a change in the shape of the whole organ. (**A**) Top: Layer-specific imaging of the developing gut (early stages to the left, more developed ones to the right). Endodermal cells are initially elongated along the circumferential direction, but they change their shape during organ folding. (**B**) Three-dimensional representation of cells near the anterior fold. The aspect ratio of the endodermal cells (*a*/*b*, where *a* and *b* are the lengths of the cells in the circumferential and longitudinal directions) changes from greater than two to about one. (**C**) Hox genes regulate calcium signaling, which mediates muscle contraction (yellow cells), thus linking hox genes to organ shape through tissue mechanics. The resulting muscle contractions are mechanically coupled to the endoderm (blue), which places strain on the tissue and ultimately influences the shape of the organ.

Image credit: Adapted from Mitchell et al., 2022 (CC BY 4.0).

Modeling the gut epithelium as an incompressible material, they found that localized changes in the shape of cells in the gut folds accounted entirely for both folding and extending of the organ. In other words, gut constrictions simultaneously converge the tissue circumferentially and extend the tissue longitudinally. Mitchell et al. term this new morphological mechanism “convergent extension via constriction”.

Based on prior work, the researchers hypothesized that localized muscle contractions by the outer layer of the gut could provide the force necessary for the gut to constrict (Bilder and Scott, 1995; Wolfstetter et al., 2009). To test this idea, they employed optogenetic tools to either inhibit or stimulate muscle contractions at specific positions along the gut tube. Strikingly, they found that localized muscle contractions were both necessary and sufficient for gut constriction. Both gain or loss of muscle constrictions led to defects in the shapes of the underlying epithelial cells and in the folding of the organ.

These data provide a clear and convincing example of one tissue layer exerting both mechanical force and morphological change onto another layer. But if gut contortions are ultimately genetically encoded, what molecular information drives muscle contractions at precise positions? The homeotic (hox) transcription factors *Antp* and *Ubx* are known regulators of organ shape and are expressed at various positions along the muscle layer (Tremml and Bienz, 1989). *Antp* mutants lack the anterior gut fold, while *Ubx* mutants lack the central fold, suggesting these patterned transcription factors could promote contractions in local muscle cells. Using high-speed calcium imaging as a proxy for muscle contraction, Mitchell et al. found that calcium pulses in the muscle layer concentrated at the positions of all three folds (Figure 2C). Moreover, localized calcium pulses were lost in *Antp* mutants.

The results of this study demonstrate that regional hox gene expression promotes calcium signaling and muscle contractions at precise positions in the developing gut. Further, they show how mechanical coupling between layers of tissue both folds and extends the tissue into stereotyped contortions. These findings add to the growing body of research emphasizing the importance of smooth muscle as a sculptor of epithelial organs, such as the vertebrate gut and mammalian lung (Shyer et al., 2013; Huycke et al., 2019; Jaslove and Nelson, 2018). The advances in deep-tissue imaging and image analysis open new possibilities for in toto imaging of a vast variety of internal organs. Moreover, they provide a framework for evaluating how adjacent tissue layers may mechanically interact.
